# Craniofacial Morphology and Foot Posture: Prevalence of Foot and Postural Deviations and Their Association with Sagittal Skeletal Class in a Cross-Sectional Clinical Study

**DOI:** 10.3390/jcm15187186

**Published:** 2026-09-16

**Authors:** Santiago Beltrán-Megías, Isabel Carda-Navarro, Lidia Galán-López, Esther García-Miralles, Clara Guinot-Barona, Laura Marqués-Martínez, Paula Iborra Plaja, Juan Ignacio Aura-Tormos

**Affiliations:** 1 Dentistry Department, Medicine and Health Science Faculty, Catholic University of Valencia, 46001 Valencia, Spain; santiago.beltran@mail.ucv.es (S.B.-M.); isabel.carda.18@mail.ucv.es (I.C.-N.); lidia.galan@ucv.es (L.G.-L.); laura.marques@ucv.es (L.M.-M.); paipla@mail.ucv.es (P.I.P.); 2 Stomatology Department, University of Valencia, 46010 Valencia, Spain; m.esther.garcia@uv.es (E.G.-M.); juan.aura@uv.es (J.I.A.-T.)

**Keywords:** cephalometry, craniofacial morphology, foot posture index, posture, sagittal skeletal class

## Abstract

**Objectives:** To describe foot and global postural deviations in a mixed-age university-clinic sample and examine their associations with cephalometrically defined sagittal skeletal class and facial growth pattern. **Methods:** This cross-sectional study included 98 individuals aged 8–62 years; 76 participants (77.6%) were adults. Foot posture was assessed with the FPI-6 and plantar footprint, global posture by sagittal-plane visual analysis, and centre-of-pressure behaviour by instrumented gait in a subsample. Associations were tested using chi-square or Fisher–Freeman–Halton exact tests, Cramér’s V, and Bonferroni-adjusted *p*-values. Age- and sex-adjusted continuous modelling was not possible because complete data could not be reliably reconstructed. **Results:** Supination was the most frequent FPI-6 category (45.9%), and 30.8% showed ideal alignment. Sagittal skeletal class was not associated with any outcome (all *p* ≥ 0.341; all pBonf = 1.000). Facial pattern was associated with FPI-6 in the primary analysis (exact *p* = 0.007; V = 0.265), but not after Bonferroni correction (pBonf = 0.070). The five-category sensitivity analysis remained significant (exact *p* = 0.0016; pBonf = 0.016; V = 0.353), although intermediate groups were small. **Conclusions:** No detectable association was found between sagittal skeletal class and foot, postural, or gait variables. The facial-pattern finding remains exploratory, and the lack of age and sex adjustment limits inference.

## 1. Introduction

Postural control integrates visual, vestibular, and proprioceptive inputs [1]. Neuroanatomical interactions between trigeminal afferents and postural control provide biological plausibility for a possible craniofacial–postural link [2,3], and sagittal jaw discrepancies have been associated with compensatory head positioning [4]. However, biological plausibility should not be interpreted as evidence of an established clinical pathway. The available literature is heterogeneous, and no predictable descending causal chain from craniofacial morphology to distal foot posture has been demonstrated [5]. Acute experimental studies of occlusal manipulation have reported immediate postural changes [6,7], whereas clinical cross-sectional studies under habitual conditions are frequently inconsistent or negative [8].

At the distal end of the kinetic chain, the foot is the primary sensorimotor interface with the ground, and its static posture can be characterised using validated tools such as the FPI-6 and plantar footprint analysis [9,10]. Reported associations between dental or craniofacial variables and plantar or postural findings have generally been weak, inconsistent, and dependent on the methods and populations studied [11,12].

Age and developmental status may modify these relationships. Craniofacial morphology, plantar-arch characteristics, and postural control continue to change during childhood and adolescence; consequently, findings in growing participants cannot necessarily be extrapolated to skeletally mature adults. Mixed-age studies therefore require explicit consideration of age as a potential confounder and, where possible, analyses restricted to mature participants.

Accordingly, the descriptive aim of this study was to characterise the prevalence of foot and global postural deviations in a mixed-age university-clinic sample. The principal inferential question was whether cephalometrically defined sagittal skeletal class was associated with static foot posture, plantar footprint morphology, global postural patterns, or gait-related centre-of-pressure behaviour. Analyses involving facial growth pattern were treated as exploratory. The study was framed as an association-screening investigation and not as a test of a presumed causal relationship.

## 2. Materials and Methods

### 2.1. Design and Reporting

This observational, cross-sectional study was designed and reported in accordance with the STROBE statement [13]. Data were collected over two academic years following an identical examination protocol and merged into a single analytical dataset. The study followed the Declaration of Helsinki and was approved by the Ethics Committee of the Universidad Católica de Valencia San Vicente Mártir under approval number UCV/2021-2022/200 (initial approval: 21 July 2022). A subsequent amendment extending the study was approved by the same Ethics Committee on 28 January 2025. The Committee confirmed that the amendment did not involve any substantial modifications to the original project. All data included in the present study were collected and processed in accordance with the original approved protocol and its subsequent amendment. All participants, or their legal guardians in the case of minors, provided written informed consent.

### 2.2. Participants

Participants were individuals consecutively assessed at the University Dental Clinic of the Universidad Católica de Valencia San Vicente Mártir (UCV), within the postgraduate programmes in Paediatric Dentistry and Orthodontics. Recruitment took place during the 2022–2023 and 2024–2025 academic years (September to June), following the initial ethical approval granted in July 2022. Potential participants attended the general dental service directly or were referred from other clinical units or postgraduate programmes for orthodontic assessment, generally because of suspected malocclusion or another orthodontic indication.

Eligible participants were patients referred for or requesting an orthodontic diagnostic assessment who had not previously undergone orthodontic treatment and who were able to complete the core craniofacial and foot assessments. Availability of valid global-posture and instrumented-gait data was not an eligibility requirement. All study measurements were obtained before the initiation of orthodontic treatment. Exclusion criteria were previous orthodontic treatment, craniofacial malformations or developmental anomalies, previous maxillofacial surgery, major orthopaedic surgery, neurological disorders, or vestibular disorders, and any condition preventing independent standing or walking.

A total of 107 individuals were assessed for eligibility. Eight did not meet the eligibility criteria: seven because of previous orthodontic treatment and one because of previous maxillofacial surgery. No eligible individual declined participation; therefore, 99 participants were enrolled and assessed. One enrolled participant was subsequently excluded because of incomplete core craniofacial or FPI-6 data.

The analytical sample comprised 98 individuals (52 men, 46 women) aged 8–62 years: 5 (5.1%) were younger than 12 years, 17 (17.3%) were aged 12–17 years, and 76 (77.6%) were 18 years or older. None had a history of orthodontic treatment, major orthopaedic surgery, or vestibular disorders. Because the sample combined growing and skeletally mature individuals, age was treated as a relevant interpretive factor. Participant flow and outcome-specific analytical samples are summarised in Supplementary Figure S1.

### 2.3. Assessment Protocol and Staged Availability

Because the diagnostic assessments had different technical requirements, the available sample varied by analysis: complete craniofacial and bilateral FPI-6 and plantar footprint data were available for 98 participants; standardised global postural assessment data were available for 91, with seven participants excluded because the postural images did not meet the required quality criteria; and instrumented gait data were available (CoP) for 57, with 41 participants excluded because their recordings were incomplete or did not meet the validity criteria. These outcome-specific reductions resulted from technical, logistical, or data-quality factors and were not based on participants’ age, sex, craniofacial classification, or other clinical characteristics. Analyses were performed on complete cases within each domain, without imputation.

Static foot posture was measured barefoot in relaxed bipedal stance with the FPI-6 (range −12 to +12); both feet were assessed independently and the arithmetic mean of the right- and left-foot FPI-6 scores was calculated to obtain a single participant-level value. This approach avoided treating measurements from the same participant as independent observations. For the categorical analyses, the bilateral mean was classified as supinated (mean < 0), neutral (mean 0 to +5), or pronated (mean > +5), following the same decision boundaries described by Redmond et al. for individual-foot scores [9].

Plantar footprint morphology was classified as cavus, normal or flat (Hernández-Corvo protocol). Global posture was evaluated by standardised sagittal-plane visual analysis, recording forward head posture, lumbar hyperlordosis and thoracic hyperkyphosis. Instrumented gait used a pressure platform (FDM system; Zebris Medical GmbH, Isny, Germany) during barefoot walking at a self-selected speed; CoP displacement was categorised in the anteroposterior and mediolateral axes [10]. Skeletal class (SNA, SNB, ANB) and facial growth pattern (facial axis, mandibular plane, saddle–articular–gonial angle sum) were determined from lateral cephalometric radiographs by calibrated examiners. The two intermediate vertical categories were collapsed into their nearest canonical pattern before any associations were examined and without inspection of the outcome distributions (meso-brachyfacial into brachyfacial; meso-dolichofacial into dolichofacial), yielding brachyfacial (*n* = 53), mesofacial (*n* = 20) and dolichofacial (*n* = 25).

The podiatric, postural, and craniofacial assessments were conducted as part of the initial diagnostic process, although they were not necessarily performed on the same day. The examiners responsible for each assessment domain were unaware of the results obtained in the other domains.

### 2.4. Reliability and Statistical Analysis

Two examiners were calibrated against an experienced clinician. Inter- and intra-examiner reliability was evaluated in a subsample of 20 participants. Inter-examiner agreement for categorical variables ranged from κ = 0.78 to 0.89 and intra-examiner agreement from κ = 0.82 to 0.93; intraclass correlation coefficients for continuous measurements ranged from 0.88 to 0.96. Categorical variables were summarised as frequencies and percentages. Associations were examined with the Pearson chi-square test; when more than 20% of cells had expected counts below five, the Fisher–Freeman–Halton exact test was used and reported alongside. Cramér’s V was used as the effect-size measure and Pearson standardised residuals (±1.96) were inspected.

The principal inferential question concerned the five associations between sagittal skeletal class and the foot, postural, and gait outcomes. The five analyses involving facial growth pattern were considered exploratory. To account for multiple comparisons, Bonferroni-adjusted *p*-values were additionally calculated across all 10 categorical association tests as pBonf = min(10 × *p*, 1). Both unadjusted and adjusted *p*-values are reported.

No formal a priori sample-size calculation was performed. The study size was determined by the number of eligible individuals available during the recruitment periods, all of whom were invited to participate. The detectable-effect calculations were conducted solely to contextualise the statistical resolution of the available sample and were not used to determine its size.

As a sensitivity analysis, the five associations involving facial growth pattern were repeated using the original five-category classification. Because of sparse cells, Fisher–Freeman–Halton exact tests were used. These sensitivity analyses were considered exploratory, and Bonferroni-adjusted *p*-values were calculated using the same 10-test correction applied to the principal categorical analyses.

To aid the interpretation of null findings, we computed the smallest effect the design could reliably detect (α = 0.05, power = 0.80): for the 3 × 3 tables (*n* = 98) this corresponded to Cramér’s V ≈ 0.25, for the global-posture analysis (*n* = 91) to V ≈ 0.29, and for the gait subsample (*n* = 57) to V ≈ 0.32. The absence of an association is therefore interpreted as absence of a detectable effect of at least this magnitude, not as proof of no association. Statistical significance was set at *p* < 0.05 for unadjusted analyses and at pBonf < 0.05 after family-wise correction; analyses were performed in JASP (version 0.19) by J.I.A.-T., a university professor and study author.

Although an age- and sex-adjusted analysis of the bilateral mean FPI-6 score was considered, complete continuous scores and covariate data could not be reliably reconstructed for the entire pooled sample. Consequently, adjusted continuous modelling and the adult-only sensitivity analysis were not performed. Analyses restricted to the available subset were avoided because of the potential for selection bias.

### 2.5. Use of Generative Artificial Intelligence Tools

During revision, OpenAI Codex (GPT-5; OpenAI; San Francisco, CA, USA; accessed 21 June 2026) was used to assist with organising reviewer comments and drafting English-language revisions. Claude (Sonnet 5; Anthropic; San Francisco, CA, USA; accessed 21 June 2026) had previously been used for English-language editing. Neither tool was provided with raw participant data or used to perform the statistical analyses. All outputs, references, analyses, and scientific interpretations were independently checked, revised, and approved by the authors, who take full responsibility for the final content.

## 3. Results

### 3.1. Sample and Prevalence of Foot and Postural Deviations

The sample comprised 98 participants (52 men, 46 women). Sagittal skeletal Class II was most prevalent (41.8%), followed by Class I (38.8%) and Class III (19.4%); facial growth pattern was distributed as brachyfacial (54.1%), dolichofacial (25.5%) and mesofacial (20.4%) (Table 1). Foot and postural deviations were common. On the FPI-6, supination was the most frequent category under Redmond’s normative cut-offs (45.9%, *n* = 45), followed by neutral (34.7%, *n* = 34) and pronation (19.4%, *n* = 19). In the postural subsample (*n* = 91), only 30.8% (*n* = 28) presented ideal alignment; lumbar hyperlordosis was the most frequent deviation (37.4%), followed by combined lumbar hyperlordosis and thoracic hyperkyphosis (13.2%), forward head posture (11.0%) and thoracic hyperkyphosis (7.7%) (Table 2).

### 3.2. Skeletal Class and Foot, Postural and Gait Variables

Sagittal skeletal class was not significantly associated with any foot, postural or gait variable: FPI-6 category (χ^2^[4] = 4.11, *p* = 0.391; V = 0.145), footprint morphology (χ^2^[4] = 3.74, *p* = 0.442; V = 0.138), global postural pattern (χ^2^[8] = 9.01, *p* = 0.341; V = 0.223), and anteroposterior (χ^2^[4] = 1.80, *p* = 0.773; V = 0.126) or mediolateral CoP displacement (χ^2^[4] = 3.39, *p* = 0.495; V = 0.172) (Table 3). All observed effect sizes lay below the smallest effect the design could reliably detect (V ≈ 0.25–0.32), so these nulls indicate the absence of a detectable moderate association rather than the exclusion of any relationship.

### 3.3. Facial Growth Pattern and Plantar Support

**Figure 1:**
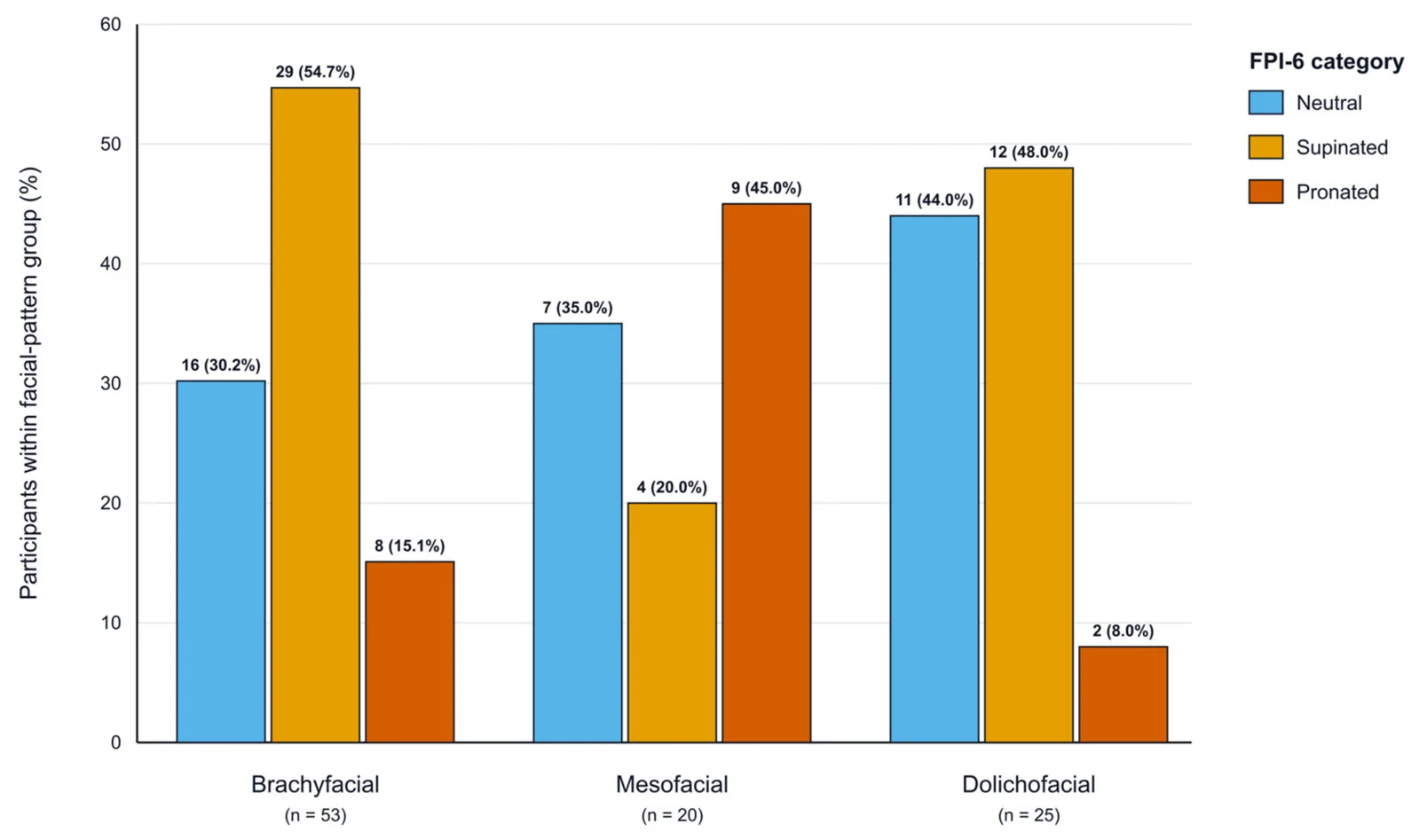
Distribution of FPI-6 categories according to the primary three-category facial growth-pattern classification (*n* = 98). Bars represent percentages within each facial-pattern group; data labels report *n* (%). FPI-6, six-item Foot Posture Index.

In exploratory analysis, facial growth pattern was associated with FPI-6 category (χ^2^[4] = 13.72, Pearson *p* = 0.008; Fisher–Freeman–Halton *p* = 0.007; Cramér’s V = 0.265). However, this association did not meet the Bonferroni-adjusted threshold (pBonf = 0.070). Mesofacial individuals showed the highest prevalence of pronated foot posture (45.0%, 9/20), whereas brachyfacial and dolichofacial individuals were more frequently classified as supinated (54.7%, 29/53, and 48.0%, 12/25, respectively; Figure 1). Complete cell counts and within-group percentages are displayed in Figure 1. The distribution was non-monotonic, with the intermediate mesofacial group differing from both extreme patterns. The parallel association with footprint morphology was also not statistically significant after adjustment (Fisher–Freeman–Halton *p* = 0.068; pBonf = 0.680; Cramér’s V = 0.210). These findings are therefore considered exploratory and hypothesis-generating.

In the sensitivity analysis retaining the original five facial-pattern categories, the association with FPI-6 remained significant (Fisher–Freeman–Halton *p* = 0.0016; pBonf = 0.016; Cramér’s V = 0.353). The association with footprint morphology was nominally significant but did not remain significant after correction (exact *p* = 0.018; pBonf = 0.183; Cramér’s V = 0.298). Associations with global posture and anteroposterior or mediolateral CoP displacement remained non-significant (all exact *p* ≥ 0.611; Cramér’s V ≤ 0.238). Given the small sizes of the intermediate facial-pattern groups, these sensitivity findings remain exploratory.

## 4. Discussion

This study described the prevalence of foot and postural deviations in a mixed-age university-clinic sample and tested their association with craniofacial morphology. The study yielded two principal observations and one exploratory finding. First, foot and postural deviations were common, with supination being the most frequent FPI-6 category and fewer than one-third of participants showing ideal global alignment. Second, these deviations were not detectably associated with sagittal skeletal class across any foot, plantar, postural or gait outcome. Third, an exploratory association between facial growth pattern and static foot posture emerged, but it was statistically fragile and biologically difficult to interpret.

### 4.1. A Common but Skeletal-Class-Independent Burden of Deviations

The high frequency of foot and postural deviations, uncoupled from skeletal class, is consistent with cross-sectional work reporting no clinically relevant relationship between natural occlusion and static posture [8], and with reviews concluding that no predictable occlusion–posture relationship can be demonstrated [14,15]. Such deviations are common even in asymptomatic individuals and are influenced by age, physical activity and sedentary behaviour rather than occlusal features alone [16,17]. Their prevalence, independent of skeletal class, reinforces the view that foot and global posture are multifactorial and should be assessed on their own terms rather than inferred from craniofacial findings.

The absence of association with skeletal class should be read within the limits of statistical resolution. With the available sample, the design could reliably exclude only associations of Cramér’s V ≥ approximately 0.25; the observed skeletal-class effect sizes (V = 0.13–0.22) all fell below this bound. The correct inference is therefore the absence of a detectable moderate association, not proof that no association of any size exists. Accordingly, foot or postural findings should not be attributed to cephalometrically defined sagittal skeletal class on the basis of these data.

### 4.2. The Facial-Pattern Signal: Exploratory and Fragile

Facial growth pattern showed an exploratory association with FPI-6 in the primary three-category analysis, although it did not meet the Bonferroni-adjusted threshold. The sensitivity analysis retaining the original five facial-pattern categories strengthened this association, which remained significant after correction. This indicates that the finding was not solely produced by collapsing the intermediate categories. Nevertheless, the distribution remained non-monotonic, and the mesobraquifacial and mesodolicofacial groups contained only 10 and 6 participants, respectively. The nominal association with footprint morphology in the five-category sensitivity analysis did not remain significant after correction, while the postural and CoP findings remained null. Therefore, the facial-pattern finding remains exploratory and requires confirmation in larger samples with adequately represented facial-pattern categories.

### 4.3. Age Heterogeneity as an Interpretive Key

A central feature of this sample is its broad age range (8–62 years), including a substantial proportion of still-growing individuals. Some studies in children and adolescents have reported associations between craniofacial features and postural or podal variables [5,18,19,20], although the evidence remains heterogeneous and does not establish that developing populations are more responsive to craniofacial inputs than adults.

Physical activity may provide an additional source of between-participant variability. Repetitive forefoot loading in activities such as ballet has been associated with greater first-ray mobility, although no correlation with hours of ballet or en-pointe practice was observed [21]. This evidence does not demonstrate a craniofacial–foot pathway; rather, it illustrates how activity-specific loading, technical execution, inherited foot morphology, and local foot mechanics may influence plantar outcomes independently of craniofacial classification.

### 4.4. Implications for Clinical Practice

For dentists, orthodontists and other clinicians, the practical message is twofold: foot and postural deviations are common in this mixed-age university-clinic sample, and they are not detectably predicted by sagittal skeletal class. The present findings do not support routinely inferring a patient’s cephalometrically defined sagittal skeletal class from foot posture, or recommending dental, orthodontic, or podiatric referral solely on the basis of one of these findings. Referral may nevertheless be appropriate when independently indicated by the patient’s symptoms or clinical examination. The exploratory facial-pattern signal is insufficient to justify incorporating craniofacial type into routine foot-posture assessment. Given the convenience sampling and the wide, unstratified age range, these findings should not be generalised beyond comparable mixed-age clinical populations until confirmed in age-stratified samples.

### 4.5. Limitations

The cross-sectional design precludes causal inference. No longitudinal follow-up was available to evaluate temporal or developmental changes within participants. Recruitment was based on a consecutive convenience sample from a single university clinic, which may have introduced selection bias and limits generalisability. The wide age range could not be analysed reliably according to developmental stage, leaving developmental confounding unresolved. The study was not designed as an equivalence trial; the negative findings should be read as the absence of a detectable, clinically relevant relationship rather than proof that no association exists, and the study had 80% power only for effects of approximately Cramér’s V = 0.25–0.32, depending on the analysis. The global-posture and gait subsamples were smaller and correspondingly underpowered, and their results are descriptive. Physical activity level, participation in dance or other sports, training exposure, first-ray mobility, footwear, body mass, and other lower-limb biomechanical factors were not included in the analysis and may have contributed to residual confounding. Multiple categorical associations were examined. None of the primary three-category analyses met the family-wise significance threshold after Bonferroni correction; however, the facial pattern–FPI-6 association remained significant in the exploratory five-category sensitivity analysis (pBonf = 0.016). Given the small intermediate groups and sparse cells, this finding should remain hypothesis-generating. Although right- and left-foot FPI-6 scores were originally recorded and their bilateral mean was used to derive the participant-level categories, averaging both feet may have obscured clinically relevant side-to-side asymmetry. Complete continuous scores and covariate data could not be reliably reconstructed across all pooled datasets. Consequently, an age- and sex-adjusted continuous analysis could not be performed for the entire analytical sample. Performing this analysis only in the available subset could have introduced additional selection bias. Collapsing the intermediate facial-pattern categories may have influenced the estimated associations. Although the sensitivity analysis retaining the original five categories also retained the facial pattern–FPI-6 association, the small mesobraquifacial and mesodolicofacial groups resulted in sparse cells and limited the stability of this finding. Categorisation of the FPI-6 may therefore have reduced statistical sensitivity, and residual confounding by age and sex cannot be excluded.

## 5. Conclusions

Foot and postural deviations were common in this mixed-age convenience sample from a single university dental clinic, yet showed no detectable association with cephalometrically defined sagittal skeletal class across foot, plantar, global postural or gait outcomes, within a resolution that could reliably exclude moderate but not small effects. These findings do not support attributing foot posture to sagittal skeletal class or recommending cross-referral solely on that basis. The exploratory facial pattern–FPI-6 association did not meet the adjusted threshold in the primary three-category analysis but remained significant in the five-category sensitivity analysis. Given its non-monotonic distribution and the small intermediate facial-pattern groups, it should nevertheless be considered hypothesis-generating. Because the sample included growing participants and adults recruited from one clinical setting, generalisation should be restricted to comparable mixed-age clinical populations.

## Figures and Tables

**Table 1:** Demographic and craniofacial characteristics (*n* = 98).

Variable	Category	*n*	%
**Sex**	Men	52	53.1
	Women	46	46.9
**Age group**	<12 years	5	5.1
	12–17 years	17	17.3
	≥18 years	76	77.6
**Skeletal class**	Class I	38	38.8
	Class II	41	41.8
	Class III	19	19.4
**Facial pattern**	Brachyfacial	53	54.1
	Mesofacial	20	20.4
	Dolichofacial	25	25.5

Bold indicates variable headings.

**Table 2:** Global postural profile in the postural subsample (*n* = 91).

Postural Pattern	*n*	%
Ideal alignment	28	30.8
Lumbar hyperlordosis	34	37.4
Lumbar hyperlordosis + thoracic hyperkyphosis	12	13.2
Forward head posture	10	11.0
Thoracic hyperkyphosis	7	7.7

**Table 3:** Summary of all tested associations. AP, anteroposterior; CoP, centre of pressure; FPI-6, six-item Foot Posture Index. Fisher–Freeman–Halton exact *p*-values are reported for sparse contingency tables. Bonferroni-adjusted *p*-values account for all 10 categorical association tests; values greater than 1 were truncated to 1.000.

Association	*n*	χ^2^ (df)	*p*	Cramér V	Bonferroni-Adjusted *p*
Skeletal class × FPI-6	98	4.11 (4)	0.391	0.145	1.000
Skeletal class × footprint	98	3.74 (4)	0.442	0.138	1.000
Skeletal class × posture	91	9.01 (8)	0.341	0.223	1.000
Skeletal class × CoP AP	57	1.80 (4)	0.773	0.126	1.000
Skeletal class × CoP lateral	57	3.39 (4)	0.495	0.172	1.000
Facial pattern × FPI-6	98	13.72 (4)	0.007	0.265	0.070
Facial pattern × footprint	98	8.67 (4)	0.068	0.210	0.680
Facial pattern × posture	91	7.82 (8)	0.451	0.207	1.000
Facial pattern × CoP AP	57	2.58 (4)	0.630	0.150	1.000
Facial pattern × CoP lateral	57	1.97 (4)	0.741	0.132	1.000

## Data Availability

The data that support the findings of this study are available from the corresponding author upon reasonable request.

## Supplementary Materials

The following supporting information can be downloaded at: https://www.mdpi.com/article/10.3390/jcm15187186/s1. Figure S1. Participant flow.
