# Supplementary material for: Craniofacial Morphology and Foot Posture: Prevalence of Foot and Postural Deviations and Their Association with Sagittal Skeletal Class in a Cross-Sectional Clinical Study
Source: J Clin Med. 2026 Sep 16;15(18):7186. doi: 10.3390/jcm15187186 (PMC13607382; doi:10.3390/jcm15187186)
Supplement: Supplementary file 1 [file jcm-15-07186-s001.zip › jcm-4500695-supplementary.pdf]

Supplementary Figure S1. Participant flow

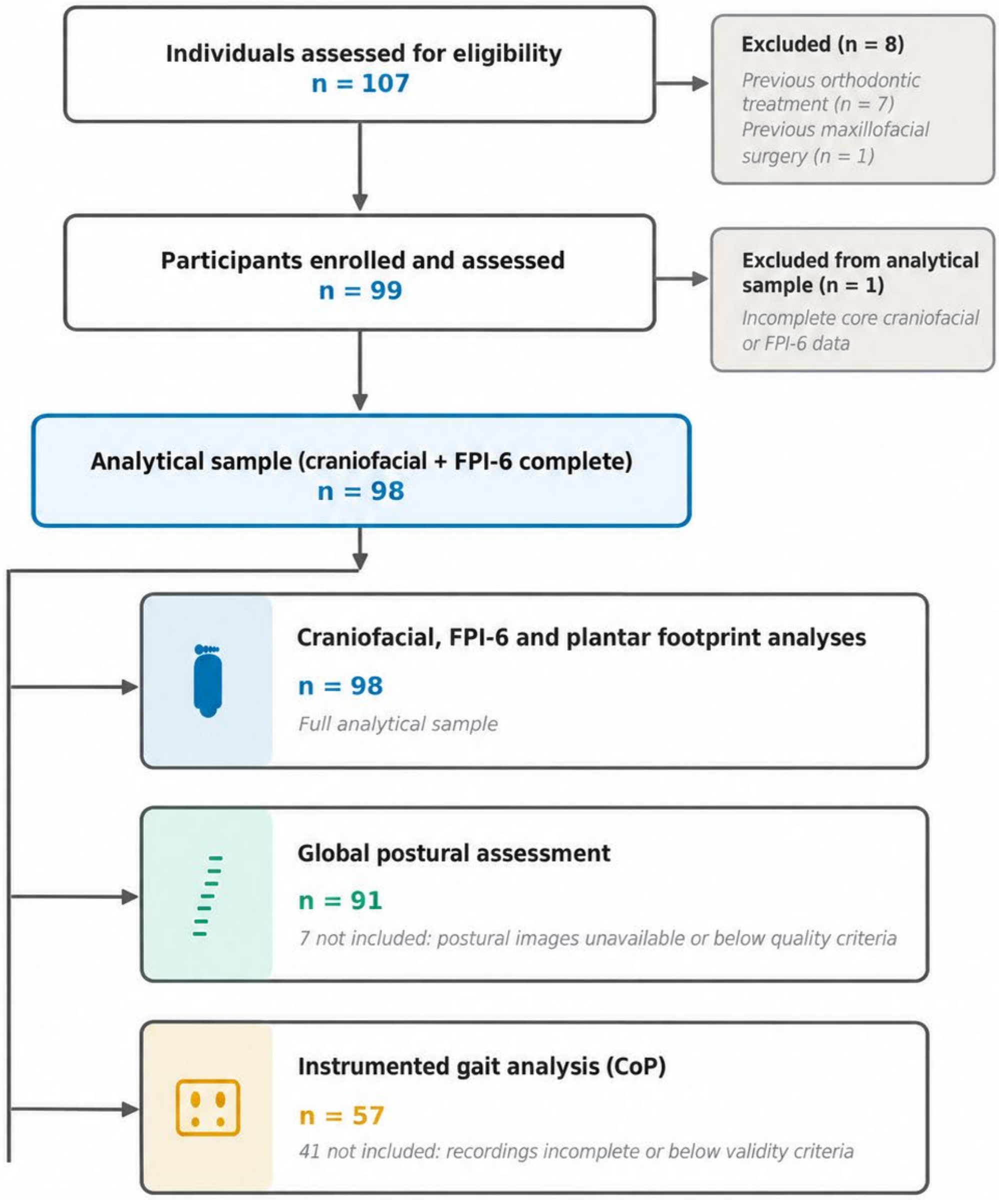

No imputation was performed; each analysis used the complete observations available for that outcome.
